# Statistical Models for High-Risk Intestinal Metaplasia with DNA Methylation Profiling

**DOI:** 10.3390/epigenomes8020019

**Published:** 2024-05-11

**Authors:** Tianmeng Wang, Yifei Huang, Jie Yang

**Affiliations:** Department of Mathematics, Statistics, and Computer Science, University of Illinois at Chicago, Chicago, IL 60607, USA; twang218@uic.edu (T.W.); yhuan39@uic.edu (Y.H.)

**Keywords:** AIC, BIC, categorical response, cross-entropy loss, cross-validation, multinomial logistic model, multinomial mixed-link model

## Abstract

We consider the newly developed multinomial mixed-link models for a high-risk intestinal metaplasia (IM) study with DNA methylation data. Different from the traditional multinomial logistic models commonly used for categorical responses, the mixed-link models allow us to select the most appropriate link function for each category. We show that the selected multinomial mixed-link model (Model 1) using the total number of stem cell divisions (TNSC) based on DNA methylation data outperforms the traditional logistic models in terms of cross-entropy loss from ten-fold cross-validations with significant *p*-values $8.12\times 10^{-4}$ and $6.94\times 10^{-5}$. Based on our selected model, the significance of TNSC’s effect in predicting the risk of IM is justified with a *p*-value less than $10^{-6}$. We also select the most appropriate mixed-link models (Models 2 and 3) when an additional covariate, the status of gastric atrophy, is available. When the status is negative, mild, or moderate, we recommend Model 2; otherwise, we prefer Model 3. Both Models 2 and 3 can predict the risk of IM significantly better than Model 1, which justifies that the status of gastric atrophy is informative in predicting the risk of IM.

## 1. Introduction

Gastric intestinal metaplasia (IM) is a precancerous change in the mucosa of the stomach with intestinal epithelium [1], which increases the risk of gastric cancer [2], the third leading cause of cancer death worldwide and the fifth most common malignancy in the world [3]. Intestinal-type gastric cancer is more common and is associated with chronic inflammation, atrophy, and IM of the stomach, often relevant to *Helicobacter pylori* infection [4]. The exact mechanism of how IM leads to gastric cancer is not fully understood, but it may involve genetic and epigenetic alterations that affect the expression and function of key genes, including DNA methylations [5]. There has been increasing evidence that DNA methylation changes in normal tissue are correlated with cancer risk [6,7,8,9,10,11,12], including gastric cancer [5,13]. The DNA methylation levels observed in IM tissue samples are significantly higher than normal gastric samples, which indicates that the DNA methylation profiles may help with predicting IM and gastric cancer [5].

In this study, we utilize the DNA methylation data of 124 samples obtained from the Gastric Cancer Epidemiology Program (GCEP) and deposited in NCBI (GSE103186) by [5]. We aim to build the most appropriate statistical model to predict the risk level of IM, including Normal (normal gastric samples), MIM (mild IM or low-risk samples, type I), and IM (high-risk samples, type II or type III), using the total number of stem cell divisions per stem cell (TNSC) estimated by the epiTOC2 (Epigenetic Timer of Cancer-2, [12]) model from the measured DNA methylation profile, along with other clinical information such as the status of gastric atrophy [5].

For categorical responses with three or more categories, such as {Normal, MIM, IM} in this study, multinomial logistic models have been widely used in the statistical literature, including the baseline-category, cumulative, adjacent-categories, and continuation-ratio logit models [14,15,16,17]. Among the four classes of logit models, the baseline-category logit model, also known as the (multiclass) logistic regression model, has been extended with a probit link and is known as the multinomial probit model [18,19,20]; the cumulative logit model has been extended to cumulative link models [19,21,22]; and the continuation-ratio logit model has been extended with a complementary log-log link [23]. It should be noted that all these models assume the same link function for all categories.

In this study, we adopt the multinomial mixed-link model (see Section 2.2), proposed by [24] recently, because it not only covers all the models mentioned above but also allows us to choose different link functions across categories. By choosing the multinomial mixed-link model, we find out that the cumulative mixed-link model with proportional odds (po) assumption and $g_{1}$ = loglog, $g_{2}$ = logit link functions outperforms the traditional models, in terms of predicting the risk level of IM using DNA methylation profiles (see Section 3.1). Based on ten-fold cross-validations, the improvement is statistically significant. Our results also show that by incorporating the status of gastric atrophy can further improve the prediction accuracy significantly. Having run our model selection procedure again, we determine that an adjacent-categories logit model with po (see Section 3.2) is most appropriate when the status of gastric atrophy is negative, mild or moderate, whereas an adjacent-categories probit model with po (see Section 3.3) works the best when the status is marked or unknown. For readers’ reference, we provide the predictive probabilities for each tissue sample in the Supplementary Materials, as well as the sample IDs and the corresponding covariates.

## 2. Materials and Methods

### 2.1. epiTOC2 Model and TNSC Covariate

The mitotic age of tissues is relevant to the total number of cell divisions, which can be estimated by the DNA methylation changes in the stem cell. Recent studies have shown the correlation between the mitotic age of tissue and the neoplastic transformation [25,26,27]. Many models for estimating mitotic age have been proposed based on DNA methylation data, including the epiTOC model [28], the solo-WCGWs model [29], and the epiTOC2 model [12]. In this study, we adopt the epiTOC2 model, which shows good robustness and is better for discriminating preneoplastic lesions [12]. The epiTOC2 model estimates the total number of stem cell divisions directly (TNSC) and is based on CpG sites marked by polycomb repressive complex-2 (PRC2). These sites are generally unmethylated across fetal tissues and become methylated during ontogeny and aging. The epiTOC2 model was fitted using the Illumina Infinium 450k data from [30], who selected $n_{c}=163$ CpG sites in their model based on the rate of increase in DNA methylation rates. A simplified epiTOC2 model can be rewritten as a weighted average of DNA methylation beta values over the $n_{c}$ CpGs in a sample *s* as follows:

$$\mathrm{TNSC}\left(s\right)=\frac{1}{n_{c}}\sum_{i=1}^{n_{c}}w_{i}\beta_{is}=\frac{1}{n_{c}}\sum_{i=1}^{n_{c}}\frac{2\beta_{is}}{\delta_{i}}$$

where $\delta_{i}$ is a model parameter representing the probability of de novo methylation of parent and daughter strands (see [12] for more details).

In this study, we first use TNSC as the only covariate representing the DNA methylation profile to predict the risk level of IM (see Section 3.1).

### 2.2. Multinomial Mixed-Link Models

In general, we consider *d* covariates or predictors with *m* distinct settings $x_{i}={\left(x_{i1},\ldots ,x_{id}\right)}^{T}$, for i=1,…,m. At the *i*th setting, $n_{i}$ categorical responses are collected and summarized into a multinomial response $Y_{i}={\left(Y_{i1},\cdots ,Y_{iJ}\right)}^{T}∼\mathrm{Multinomial}(n_{i};\pi_{i1},\cdots ,$$\pi_{iJ})$, where $Y_{ij}$ is the number of observations with the *j*th response category, and $\pi_{ij}$ is the probability that the response falls into the *j*th category, j=1,…,J. Assuming all $\pi_{ij}\in \left(0,1\right)$, there are four classes of multinomial logit models that have ever been used in the literature (see [16] and the references therein):

(1) $$\begin{array}{ccc} \log\left(\frac{\pi_{ij}}{\pi_{iJ}}\right) & = & \beta_{0j}+\beta_{j}^{T}x_{i},\;\mathrm{baseline}-\mathrm{category} \end{array}$$

(2) $$\begin{array}{ccc} \log\left(\frac{\pi_{i1}+\cdots +\pi_{ij}}{\pi_{i,j+1}+\cdots +\pi_{iJ}}\right) & = & \beta_{0j}+\beta_{j}^{T}x_{i}\;,\;\mathrm{cumulative} \end{array}$$

(3) $$\begin{array}{ccc} \log\left(\frac{\pi_{ij}}{\pi_{i,j+1}}\right) & = & \beta_{0j}+\beta_{j}^{T}x_{i}\;,\;\mathrm{adjacent}-\mathrm{categories} \end{array}$$

(4) $$\begin{array}{ccc} \log\left(\frac{\pi_{ij}}{\pi_{i,j+1}+\cdots +\pi_{iJ}}\right) & = & \beta_{0j}+\beta_{j}^{T}x_{i},\;\mathrm{continuation}-\mathrm{ratio} \end{array}$$

where $\beta_{j}={\left(\beta_{j1},\ldots ,\beta_{jd}\right)}^{T}$, i=1,…,m, and j=1,…,J−1. In the statistical literature (see, for example, [16]), the four logit models, (1)–(4), are also called nonproportional odds (npo) models, which allow $\beta_{j}$’s to be different across j=1,…,J−1. If we further assume $\beta_{j}\equiv \beta ={\left(\beta_{1},\ldots ,\beta_{d}\right)}^{T}$, then the four models are known as proportional odds (po) models. For more general odds structures for multinomial logistic models, that is, partial proportional odds (ppo) models, please see [16,17].

In the form of npo models, the multinomial mixed-link model [24] can be written as follows

(5) $$g_{j}\left(\rho_{ij}\right)=\beta_{0j}+\beta_{j}^{T}x_{i}$$

where

(6) $$\rho_{ij}=\left\{\begin{array}{cc} \frac{\pi_{ij}}{\pi_{ij}+\pi_{iJ}} & ,\mathrm{for}\;\mathrm{baseline}-\mathrm{category}\;\mathrm{mixed}-\mathrm{link}\;\mathrm{models}\\ \pi_{i1}+\cdots +\pi_{ij} & ,\mathrm{for}\;\mathrm{cumulative}\;\mathrm{mixed}-\mathrm{link}\;\mathrm{models}\\ \frac{\pi_{ij}}{\pi_{ij}+\pi_{i,j+1}} & ,\mathrm{for}\;\mathrm{adjacent}-\mathrm{categories}\;\mathrm{mixed}-\mathrm{link}\;\mathrm{models}\\ \frac{\pi_{ij}}{\pi_{ij}+\cdots +\pi_{iJ}} & ,\mathrm{for}\;\mathrm{continuation}-\mathrm{ratio}\;\mathrm{mixed}-\mathrm{link}\;\mathrm{models} \end{array}\right.$$

where $g_{j}$ is a predetermined link function, i=1,…,m, and j=1,…,J−1. It can be verified that if $g_{1}\left(\rho_{ij}\right)\equiv \cdots \equiv g_{J-1}\left(\rho_{ij}\right)=\log\left(\rho_{ij}/\left(1-\rho_{ij}\right)\right)$, that is, the logit link, then the multinomial mixed-link model (5) plus (6) leads to the four multinomial logit models (1)–(4). In this study, we also consider some other link functions that have been commonly used in the literature, namely, probit ($g_{j}\left(\rho_{ij}\right)=\Phi^{-1}\left(\rho_{ij}\right)$, where Φ is the cumulative distribution function of standard normal distribution), log-log (or loglog, $g_{j}\left(\rho_{ij}\right)=-\log\left(-\log\left(\rho_{ij}\right)\right)$, and complementary log-log (or cloglog, $g_{j}\left(\rho_{ij}\right)=\log\left(-\log\left(1-\rho_{ij}\right)\right)$. For more options of link functions, please see Table 1 in [24].

Following the notation in [24], the multinomial mixed-link model (5) plus (6) can be written into its matrix form:

(7) $$g\left(\frac{L\pi_{i}}{R\pi_{i}+\pi_{iJ}b}\right)=\beta_{0}+B^{T}x_{i}$$

where $g={\left(g_{1},\ldots ,g_{J-1}\right)}^{T}$, L and R are (J−1)×(J−1) constant matrices, b is a constant vector of length J−1, $\pi_{i}={\left(\pi_{i1},\ldots ,\pi_{i,J-1}\right)}^{T}$, $\pi_{iJ}=1-\sum_{j=1}^{J-1}\pi_{ij}$, $\beta_{0}={\left(\beta_{01},\ldots ,\beta_{0,J-1}\right)}^{T}$, $B=(\beta_{1},\ldots ,\beta_{J-1})$ is a d×(J−1) matrix of parameters. Note that the vector g of link functions in (7) applies to the ratio of two vectors component-wise. That is, if we denote $L={\left(L_{1},\ldots ,L_{J-1}\right)}^{T}$, $R={\left(R_{1},\ldots ,R_{J-1}\right)}^{T}$ and $b={\left(b_{1},\ldots ,b_{J-1}\right)}^{T}$, then the multinomial mixed-link model (7) can be written in its equation form:

$$g_{j}\left(\frac{L_{j}^{T}\pi_{i}}{R_{j}^{T}\pi_{i}+\pi_{iJ}b_{j}}\right)=\beta_{0j}+\beta_{j}^{T}x_{i}\;,\;j=1,\ldots ,J-1$$

In other words, $\rho_{ij}$ in (5) and (6) can be written as

$$\rho_{ij}=\frac{L_{j}^{T}\pi_{i}}{R_{j}^{T}\pi_{i}+\pi_{iJ}b_{j}},\;j=1,\ldots ,J-1$$

In this study, we consider the four classes of mixed-link models listed in (6). For baseline-category mixed-link models, $L=R=I_{J-1}$, the identity matrix of order J−1, and $b=1_{J-1}$, the vector of ones with length J−1; for cumulative mixed-link models,

$$L=\left[\begin{array}{cccc} 1 & & & \\ 1 & 1 & & \\ \vdots & \vdots & \ddots & \\ 1 & 1 & \cdots & 1 \end{array}\right]\;\in R^{\left(J-1)\times (J-1\right)}$$

$R=1_{J-1}1_{J-1}^{T}$, and $b=1_{J-1}$; for adjacent-categories mixed-link models, $L=I_{J-1}$,

$$R=\left[\begin{array}{ccccc} 1 & 1 & & & \\ & 1 & 1 & & \\ & & 1 & \ddots & \\ & & & \ddots & 1\\ & & & & 1 \end{array}\right]\in R^{\left(J-1)\times (J-1\right)},\mathrm{and}\;b=\left[\begin{array}{c} 0\\ 0\\ \vdots \\ 0\\ 1 \end{array}\right]\in R^{J-1}$$

and for continuation-ratio mixed-link models, $L=I_{J-1}$,

$$R=\left[\begin{array}{cccc} 1 & 1 & \cdots & 1\\ & 1 & \cdots & 1\\ & & \ddots & \vdots \\ & & & 1 \end{array}\right]\in R^{\left(J-1)\times (J-1\right)}$$

and $b=1_{J-1}$.

In this study, we implement the algorithms described in Section 4 of [24] to find the maximum likelihood estimate (MLE) $\hat{\theta }$ for either the npo model’s parameter vector $\theta ={\left(\beta_{0}^{T},\beta_{1}^{T},\ldots ,\beta_{J-1}^{T}\right)}^{T}$ of length p=(d+1)×(J−1), or the po model’s $\theta ={\left(\beta_{0}^{T},\beta^{T}\right)}^{T}$ of length p=d+J−1.

### 2.3. Model Selection and Evaluation

In this study, we use the multinomial mixed-link model (5) plus (6) to predict the risk level of IM in three ordered categories, namely, Normal, MIM, and IM. In terms of the structure of $\rho_{ij}$ as defined in (6), we have four options, namely, baseline-category, cumulative, adjacent-categories, and continuation-ratio mixed-link models. In this study, the number of response categories is J=3. For each j=1,…,J−1, we consider four possible link functions, namely, logit, probit, loglog, and cloglog. From the right-hand side of (5), we still have two options, an npo model ($\beta_{0j}+\beta_{j}^{T}x_{i}$) or a po model ($\beta_{0j}+\beta^{T}x_{i}$). As a summary, we have $4\times 4^{J-1}\times 2$ candidate models.

In the statistical literature, the Akaike Information Criterion (AIC, [31,32]) and Bayesian Information Criterion (BIC, [33]) have been widely used for model selection, given that a statistical model is assumed. In our case, the maximized likelihood $l(\hat{\theta })$ is obtained along with the MLE $\hat{\theta }$ after fitting the model. In our notation,

$$\begin{array}{ccc} \mathrm{AIC} & = & -2\cdot l(\hat{\theta })+2\cdot \;p\\ \mathrm{BIC} & = & -2\cdot l\left(\hat{\theta }\right)+\log\left(n\right)\cdot p \end{array}$$

where $n=\sum_{i=1}^{m}n_{i}$ stands for the total number of observations or the sample size, p=(d+1)×(J−1) for npo models or d+J−1 for po models in our study. Smaller AIC or BIC values imply better models. Since in this study the sample size n=124 (see Section 3) is not large, we recommend AIC against BIC if their results of model selection are not consistent (see, for example, [34], for more discussions on AIC and BIC).

To show if the selected model is significantly better than commonly used models in the literature, we use a ten-fold cross-validation to estimate the prediction errors of the models under comparison. Different from five-fold cross-validations chosen by [17], we choose ten-fold cross-validations in this study because our sample size n=124 is relatively smaller (for more discussion on ten-fold versus five-fold cross-validations, see [34]).

Different from many machine learning techniques, the multinomial mixed-link model provides a stochastic classification answer [35] to each tissue sample. That is, given the covariate or predictor setting $x_{i}$, we obtain by the fitted multinomial mixed-link model predictive probabilities ${\hat{\pi }}_{ij}$ for Normal (j=1), MIM (j=2), and IM (j=3), respectively, which is much more informative than a deterministic classification answer [35]. Following [17], we use the cross-entropy loss to evaluate the performance of statistical models under comparison. Given a random partition *B* of the index set [n]={1,…,n}, which divides [n] into ten non-overlapped subsets (called blocks) of roughly the same size, the (average) cross-entropy (CE) loss for a specified model is

$$\mathrm{CE}\left(B\right)=-\frac{1}{n}\sum_{i=1}^{n}\log\left({\hat{\pi }}_{i,y_{i}}^{k(i)}\right)$$

where n=124 is the sample size, $y_{i}$ is the observed response label of the *i*th tissue sample, and k(i) is the block label to which the *i*th sample belongs. More details about calculating CE can be found in Section 2.4 of [17] except that we use a ten-fold instead of five-fold cross-validation.

A smaller CE value implies a better model. To check whether the improvement of one model against another is statistically significant, in this study we randomly generate partitions and use a one-sided paired *t*-test to check whether the improvement is significant.

## 3. Results

### 3.1. Statistical Model Selection for Predicting IM Based on TNSC

In this study, we first match the DNA methylation data downloaded from NCBI (https://www.ncbi.nlm.nih.gov/geo/, GSE103186, accessed on 23 January 2024) with the tissue samples listed in Table S3 in [5] (https://www.cell.com/cancer-cell/, accessed on 18 January 2024). Among the 134 tissue samples collected at the antrum site [5], there are 10 samples lacking DNA methylation profiles. We use the remaining 124 samples for our analysis. We then compute the TNSC values for the 124 samples using their DNA methylation data, as described in Section 2.1. The R codes for computing TNSC are accessible online (https://zenodo.org/records/2632938, epiTOC2.R, accessed on 15 January 2024) as indicated by [12]. In this section, we consider the multinomial mixed-link model as described in Section 2.2, and use the computed TNSC as the only covariate to predict the risk level of IM in three categories (Normal, MIM, and IM). For each of 4×2 models, the optimal link functions for j=1,2, respectively, along with their corresponding AIC and BIC values, are listed in Table 1 (see Appendix A for the AIC and BIC values of all link combinations).

According to Table 1, the best multinomial mixed-link model with the lowest AIC overall in this case, called Model 1, is a cumulative po model with loglog and logit links for j=1 (Normal) and j=2 (MIM), respectively. Note that by default j=3 (IM) is treated as the baseline category. The fitted Model 1 is provided in (8), where $x_{TNSC,i}$ is the computed TNSC value for the *i*th tissue sample.

(8) $$\begin{array}{c} -\log\left(-\log\left(\pi_{i1}\right)\right)=\beta_{01}+\beta_{1}x_{TNSC,i}=4.023-4.228\times 10^{-4}x_{TNSC,i}\\ \log\left(\frac{\pi_{i1}+\pi_{i2}}{\pi_{i3}}\right)=\beta_{02}+\beta_{1}x_{TNSC,i}=4.905-4.228\times 10^{-4}x_{TNSC,i} \end{array}$$

In (8), the estimated coefficient of $x_{TNSC,i}$ is $-4.228\times 10^{-4}$, which is fairly small. To test whether the effect of TNSC is significant in predicting IM, we obtain its 95% confidence interval $\left(-4.167\times 10^{-4},-4.290\times 10^{-4}\right)$, which does not contain zero. Actually, the corresponding *p*-value of its significance test is less than $10^{-6}$. As a conclusion, the effect of TNSC is statistically significant in predicting the risk level of IM.

**Figure 1:**
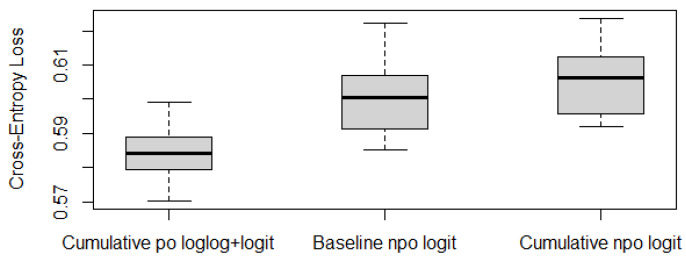
Cross-entropy loss based on ten-fold cross-validations with ten random partitions.

To further check whether Model 1 outperforms the traditional statistical models, as described in Section 2.3, we run a ten-fold cross-validation and compare its cross-entropy loss against other models. For illustration purposes, we choose the baseline-category logit model with npo (also known as the multiclass logistic regression model) and the cumulative logit model with npo (one of the most popular models for ordinal responses) as the alternative models. As for other models, including multinomial logit models and probit models, the conclusions are similar (see Appendix A). To avoid misleading conclusions relying on a particular partition, we randomly generate ten partitions and compute their corresponding CE values. The boxplots of the resulting ten CE values are provided in Figure 1, which shows that the CE values of Model 1 seem to be much lower than those values of the other two models. Although we only run ten random partitions due to computational intensity, our one-sided paired *t*-tests based on the ten CE values show that the improvements of Model 1 are significant. The *p*-values of the *t*-tests for comparing Model 1 against the baseline npo model and the cumulative npo model displayed in Figure 1 are $8.12\times 10^{-4}$ and $6.94\times 10^{-5}$, respectively. That is, the recommended cumulative po model with loglog and logit links significantly outperforms the two multinomial logistic models that are commonly used in practice.

**Figure 2:**
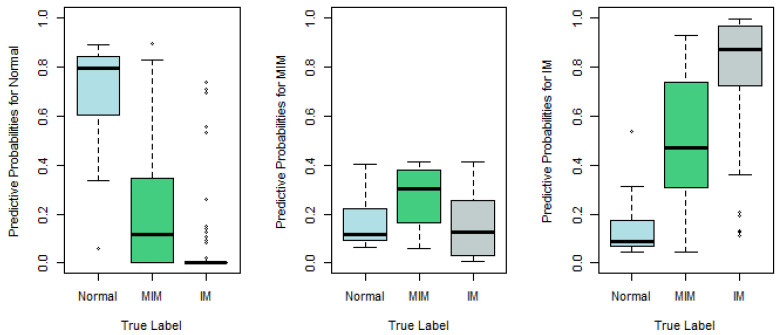
Predictive probabilities ${\hat{\pi }}_{ij}$ based on Model 1 against true response labels (left panel: j=1; middle panel: j=2; right panel: j=3).

To show how well Model 1 works, we plot in Figure 2 the predictive probabilities ${\hat{\pi }}_{ij}$ against the true response labels, j=1,2,3, respectively.

According to Figure 2, the recommended Model 1 works reasonably well. For examples, in the left panel, we plot ${\hat{\pi }}_{i1}$, which is the predictive probability that the *i*th tissue sample belongs to Normal, against its true response label. If the true label is Normal, the left boxplot in the left panel of Figure 2, which is apparently higher than the other two boxplots in the same panel, indicates that the corresponding tissue sample tends to be predicted as Normal as well. Similarly, in the right panel, ${\hat{\pi }}_{i3}$, the predictive probability that the sample belongs to IM, is plotted, and the significantly higher boxplot to the right indicates that the sample with true label IM tends to be predicted as IM as well. Nevertheless, the middle panel, which plots the predictive probabilities for MIM, indicates that the MIM class is not so different from Normal or IM, and thus is more difficult to predict correctly.

### 3.2. Statistical Model Selection for Predicting IM Based on TNSC and Gastric Atrophy

In this section, we show that when additional information, such as the status of gastric atrophy, is available, the prediction accuracy of the IM risk level can be significantly improved.

In this study, the status of gastric atrophy is a 5-class categorical variable (see Table S3 in [5]), namely, Marked, Moderate, Mild, Negative, and Unknown. In our regression analysis involving the status of gastric atrophy, we replace it with four dummy variables: $x_{mild,i}$, $x_{moderate,i}$, $x_{negative,i}$, and $x_{unknown,i}$. Each dummy variable is binary, taking a value of either 1 or 0, with at most one variable allowed to be 1 for any given sample. For instance, a configuration of $\left(x_{mild,i},x_{moderate,i},x_{negative,i},x_{unknown,i}\right)=\left(1,0,0,0\right)$ indicates a mild gastric atrophy status for the *i*th sample, (0,1,0,0) indicates a moderate gastric atrophy status, whereas (0,0,0,0) indicates a marked status, that is, the baseline status. Similarly to Table 1, we list the optimal link functions for j=1,2, respectively, along with their AIC and BIC values, in Table 2.

With the presence of gastric atrophy, the best multinomial mixed-link model, called Model 2, is an adjacent-categories logit model with po, which is different from the type of Model 1 with TNSC only (see Section 3.1). Since its AIC value, 108.89, is much less than 144.29 in Table 1, Model 2 is expected to outperform Model 1 significantly in terms of prediction accuracy (see [36] for more discussion on AIC differences). The fitted Model 2 is provided in (9).

(9) $$\begin{array}{cc} \log\left(\frac{\pi_{i1}}{\pi_{i2}}\right)= & \beta_{01}+\beta_{1}x_{TNSC,i}+\beta_{2}x_{mild,i}+\beta_{3}x_{moderate,i}+\beta_{4}x_{negative,i}+\beta_{5}x_{unknown,i}\\ = & -1.859-4.586\times 10^{-4}x_{TNSC,i}-1.144x_{mild,i}-2.103x_{moderate,i}\\ & +6.469x_{negative,i}+3.663x_{unknown,i}\\ \log\left(\frac{\pi_{i2}}{\pi_{i3}}\right)= & \beta_{02}+\beta_{1}x_{TNSC,i}+\beta_{2}x_{mild,i}+\beta_{3}x_{moderate,i}+\beta_{4}x_{negative,i}+\beta_{5}x_{unknown,i}\\ = & 0.136-4.586\times 10^{-4}x_{TNSC,i}-1.144x_{mild,i}-2.103x_{moderate,i}\\ & +6.469x_{negative,i}+3.663x_{unknown,i} \end{array}$$

**Figure 3:**
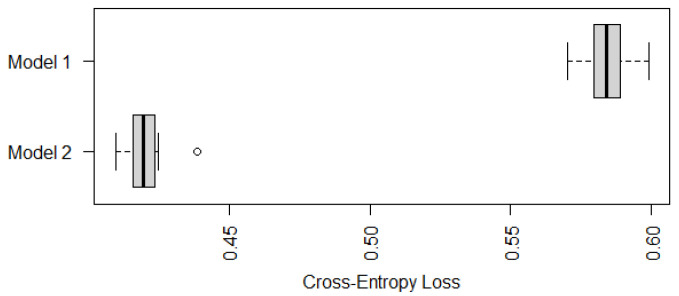
Boxplots of cross-entropy loss of Model 1 and Model 2 based on ten-fold cross-validations with ten random partitions.

Similarly to Figure 1, we compare in Figure 3 the cross-entropy loss of two recommended models shown in (8) (Model 1) and (9) (Model 2). It is not surprising that Model 2 with both TNSC and gastric atrophy as predictors has a significantly smaller cross-entropy loss, which implies that the status of gastric atrophy is informative in predicting the risk level of IM.

**Figure 4:**
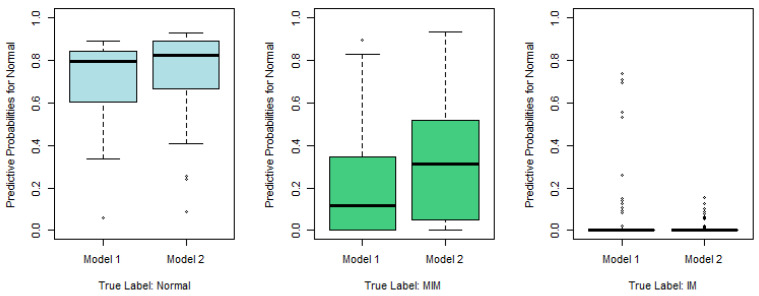
Predictive probabilities for the normal category based on Model 1 and Model 2.

**Figure 5:**
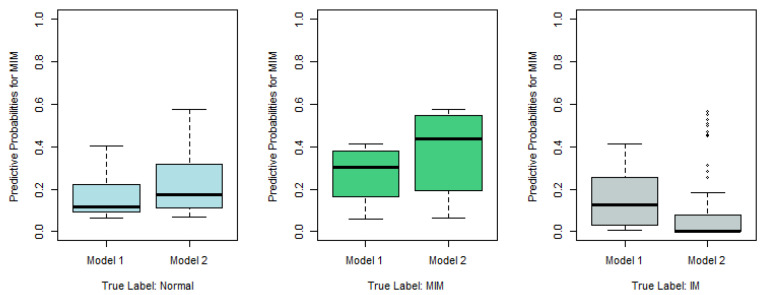
Predictive probabilities for the MIM category based on Model 1 and Model 2.

**Figure 6:**
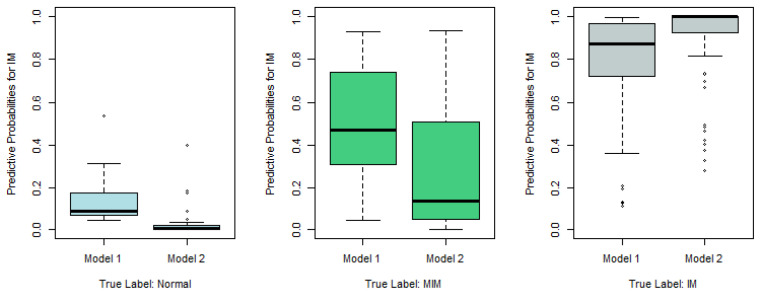
Predictive probabilities for IM category based on Model 1 and Model 2.

Similarly to Figure 2, we plot the predictive probabilities based on Model 1 and Model 2 against the true IM labels in Figure 4, Figure 5 and Figure 6. When the true IM label matches the predictive label, such as the left panel in Figure 4, the middle panel in Figure 5, and the right panel of Figure 6, Model 2 tends to provide a higher predictive probability than Model 1, which shows that overall Model 2 outperforms Model 1.

### 3.3. Statistical Model Selection after Removing Unknown and Marked Categories

Among the 124 samples considered in this study, there are only 3 cases with “Marked” status of gastric atrophy, and there are 23 cases with “Unknown” status, which is not informative. In this section, we consider the best multinomial mixed-link model for the 98 cases after removing the samples that belong to Marked or Unknown categories.

In this section, the status of gastric atrophy is a three-class categorical variable restricted to the 98 samples. Similarly to Model 2 in Section 3.2, we replace the status of gastric atrophy with two dummy variables ($x_{mild,i}$, $x_{moderate,i}$). More specifically, ($x_{mild,i}$, $x_{moderate,i}$) = (1,0) stands for mild status, (0,1) for moderate status, and (0,0) for negative status representing the baseline. Similarly to Table 1 and Table 2, we provide in Table 3 the optimal choices of link functions for each type of multinomial model. According to Table 3, the best multinomial mixed-link model for this scenario is an adjacent-categories po model with probit links for both j=1,2. We call it Model 3 and list its fitted model in (10).

(10) $$\begin{array}{cc} \Phi^{-1}\left(\frac{\pi_{i1}}{\pi_{i1}+\pi_{i2}}\right)= & \beta_{01}+\beta_{1}x_{TNSC,i}+\beta_{2}x_{mild,i}+\beta_{3}x_{moderate,i}\\ = & 3.153-3.446\times 10^{-4}x_{TNSC,i}-4.260x_{mild,i}-5.347x_{moderate,i}\\ \Phi^{-1}\left(\frac{\pi_{i2}}{\pi_{i2}+\pi_{i3}}\right)= & \beta_{02}+\beta_{1}x_{TNSC,i}+\beta_{2}x_{mild,i}+\beta_{3}x_{moderate,i}\\ = & 5.275-3.446\times 10^{-4}x_{TNSC,i}-4.260x_{mild,i}-5.347x_{moderate,i} \end{array}$$

**Figure 7:**
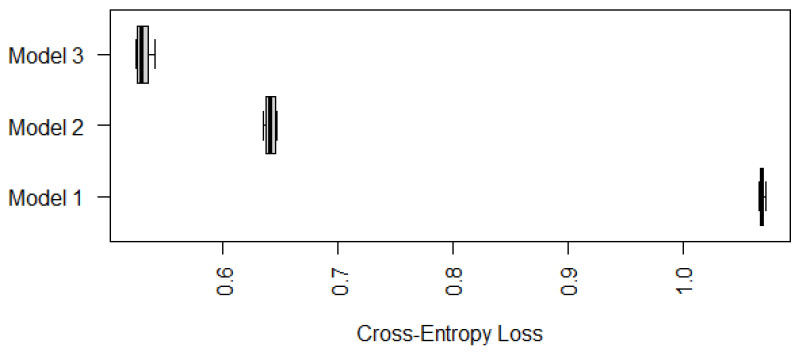
Boxplots of cross-entropy loss (on 98 Samples only) of Models 1, 2, and 3 based on ten-fold cross-validations with ten random partitions.

To compare the performance of Model 3 with Model 1 and Model 2, we use the cross-entropy loss based on ten-fold cross-validations similarly to Section 3.1 and Section 3.2. Since Model 3 cannot be applied to cases with marked or unknown status of gastric atrophy, we compare the performance of the three models on samples with mild, moderate, or negative status of gastric atrophy only. Their boxplots of cross-entropy loss based on ten random partitions for ten-fold cross-validations are displayed in Figure 7.

According to Figure 7, Model 3 has a significantly smaller (average) cross-entropy loss compared with Model 1 and Model 2, in terms of predicting IM for individuals whose gastric atrophy statuses are negative, mild or moderate. Nevertheless, Models 1 and 2 are still useful since they can be applied to cases with marked or unknown status of gastric atrophy as well.

## 4. Discussion

In Section 3, we presented three models for different scenarios. When only the TNSC (or the DNA methylation profile) is available, we recommend Model 1, a cumulative mixed-link model with po, which works reasonably well with TNSC as the only input. When the status of gastric atrophy is also available, there are two different scenarios. If the status is negative, mild, or moderate, we recommend Model 2, an adjacent-categories logit model with po, which belongs to the traditional multinomial logit models. If the status is marked or unknown, we recommend Model 3 instead, which is an adjacent-categories probit model with po. Each of the three models has its own advantages. For example, although both Model 2 and Model 3 outperform Model 1 in terms of prediction accuracy, Model 1 is still useful when the status of gastric atrophy is not available.

**Figure 8:**
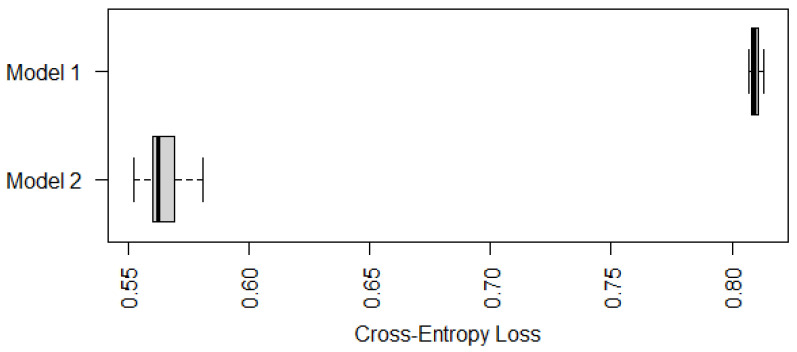
Boxplots of cross-entropy loss (on 26 Samples only) of Models 1 and 2 based on ten-fold cross-validations with ten random partitions.

To further compare the performance of Models 1 and 2 on cases with marked or unknown status of gastric atrophy, we display in Figure 8 the (average) cross-entropy loss on predicting those 26 cases with marked or unknown status of gastric atrophy only. According to Figure 8, Model 2 still outperforms Model 1 in predicting the risk level of IM for those 26 cases, which suggests that Model 2 be recommended against Models 1 and 3 for cases with marked or unknown status of gastric atrophy.

In practice, more covariates or predictors may be added to the multinomial mixed-link model as well, given their availability. For example, it is known that *Helicobacter pylori* (Hp) infection is an important factor for both IM and gastric cancer development [5,37]. When the Hp status, in terms of Hp serology test result [38], histological examination result [39], or Hp sequence reads [5], is available, one may add it into the model and use AIC, BIC, or cross-validation to determine whether the model with the newly added covariate works significantly better (see Section 2.3).

It should be noted that when using model selection techniques described in Section 2.3, sometimes the differences between the best models are not significant. For example, when selecting Model 3, two other models, a cumulative probit model with po and a continuation-ratio probit model with po, have similar AIC values (see Table 3) that are not significantly smaller than Model 3’s [36]. In this case, one may use any of them for prediction purposes. That is saying, with the current data or a finite sample size, those models are comparable or not significantly different from each other.

With an increased sample size, if there is a true statistical model associated with the response and available predictors, then the true model is expected to be among the best models asymptotically [40]. Nevertheless, it does not necessarily mean that the true model is asymptotically identifiable (see [40] for more discussion on asymptotic consistency related to model selections for multinomial models).

In a previous study [5], DNA methylation alteration has been reported as significantly correlated with IM regression at the univariate level. Nevertheless, the significance vanishes when mutation burden and Hp density are incorporated into a multivariate logistic regression analysis [5]. It is worthy of further exploration using the recommended multinomial mixed-link model with the most appropriate link functions selected.

## 5. Conclusions

In this study, we recommend the newly developed multinomial mixed-link models for predicting Intestinal Metaplasia using DNA methylation profiling. Using model selection techniques, such as AIC, BIC, and cross-validations, we show that the selected multinomial mixed-link model (Model 1) outperforms the traditional multinomial models that assume the same link function for all categories. We also show that when additional information, such as new covariates or predictors, is added to the model, the selection procedure needs to be rerun and the best mixed-link model may change.

When four or more response categories are involved, models other than multinomial mixed-link models have been proposed as well, including two-group models, which can deal with NA or unknown response categories, and po-npo mixture models, which are more flexible than npo, po, or ppo (partial proportional odds) models (see [24] for more examples). Model selection techniques described in Section 2.3 can still be applied, just to a much larger set of candidate models.

## Figures and Tables

**Table 1:** Best mixed-link models for predicting IM based on TNSC.

Model	Best Link	AIC	BIC
Baseline-category npo	loglog, loglog	146.69	157.97
Cumulative npo	loglog, probit	145.53	156.81
Adjacent-categories npo	loglog, loglog	145.97	157.25
Continuation-ratio npo	loglog, loglog	145.93	157.21
Baseline-category po	probit, logit	151.12	159.58
**Cumulative po**	**loglog, logit**	**144.29**	**152.75**
Adjacent-categories po	loglog, logit	146.33	154.79
Continuation-ratio po	loglog, logit	146.96	155.42

Note: The best model overall, along with its links and values, is highlighted in bold.

**Table 2:** Best mixed-link models for predicting IM based on TNSC and gastric atrophy.

Model	Best Link	AIC	BIC
Baseline-category npo	logit, probit	109.95	143.79
Cumulative npo	loglog, logit	109.20	143.04
Adjacent-categories npo	logit, logit	109.97	143.81
Continuation-ratio npo	logit, logit	110.97	144.82
Baseline-category po	probit, logit	111.31	131.05
Cumulative po	probit, probit	110.03	129.77
**Adjacent-categories po**	**logit, logit**	**108.89**	**128.63**
Continuation-ratio po	probit, probit	109.32	129.06

Note: The best model overall, along with its links and values, is highlighted in bold.

**Table 3:** Best mixed-link models for predicting IM based on TNSC and 3-class gastric atrophy.

Model	Best Link	AIC	BIC
Baseline-category npo	logit, probit	81.43	102.11
Cumulative npo	probit, probit	84.29	104.97
Adjacent-categories npo	logit, probit	83.22	103.90
Continuation-ratio npo	logit, probit	83.56	104.24
Baseline-category po	probit, logit	82.39	95.32
Cumulative po	probit, probit	77.99	90.92
**Adjacent-categories po**	**probit, probit**	**77.56**	**90.48**
Continuation-ratio po	probit, probit	77.77	90.69

Note: The best model overall, along with its links and values, is highlighted in bold.

## Data Availability

DNA methylation profiling data are publicly available from NCBI (GSE103186) at https://www.ncbi.nlm.nih.gov/geo/query/acc.cgi?acc=GSE103186, accessed on 23 January 2024. The clinical data are publicly available online from publication [5] Table S3 at https://www.cell.com/cancer-cell/fulltext/S1535-6108(17)30521-4, accessed on 18 January 2024.

## Supplementary Materials

The following supporting information can be downloaded at: https://www.mdpi.com/article/10.3390/epigenomes8020019/s1, Table S1: IM-epiTOC2-GA-Probabilities.csv, including 124 records with ID, Patient.ID, Response, Site, Gastric.Atrophy, TNSC, predictive probabilities for Normal (j=1), MIM (j=2) and IM (j=3) based on Model 1 (Model1_prob1, Model1_prob2, Model1_prob3), Model 2 (Model2_prob1, Model2_prob2, Model2_prob3), and Model 3 (Model3_prob1, Model3_prob2, Model3_prob3).

## Abbreviations

The following abbreviations are used in this manuscript:

AIC	Akaike information criterion
BIC	Bayesian information criterion
CE	cross entropy
CpG	5’—C—phosphate—G—3’ sequence of nucleotides
cloglog	complementary log-log link
DNA	deoxyribonucleic acid
GCEP	Gastric Cancer Epidemiology Program
ID	identifier
IM	intestinal metaplasia
loglog	log-log link
MIM	mild intestinal metaplasia
MLE	maximum likelihood estimate
npo	non-proportional odds assumption
po	proportional odds assumption
PRC2	polycomb repressive complex-2
TNSC	total number of stem cell divisions

## Appendix A. AIC and BIC Values of Multinomial Mixed-Link Models Using TNSC for Predicting IM

In this section, we provide a complete list of AIC and BIC values for the multinomial mixed-link models with link functions in {logit, probit, loglog, cloglog}. A “-” in the following tables indicates that the corresponding AIC or BIC value is not available, typically due to numerical issues when fitting the corresponding model.

**Table A1:** Baseline-category mixed-link models with npo.

	logit		probit		loglog		cloglog	
	AIC	BIC	AIC	BIC	AIC	BIC	AIC	BIC
logit	148.49	159.78	147.84	159.12	147.09	158.37	-	-
probit	148.95	160.23	148.27	159.56	147.47	158.75	-	-
loglog	148.83	160.11	148.11	159.39	**146.69**	**157.97**	-	-
cloglog	-	-	-	-	-	-	-	-

Note: The AIC/BIC values, associated with the best pair of links, are highlighted in bold.

**Table A2:** Cumulative mixed-link models with npo.

	logit		probit		loglog		cloglog	
	AIC	BIC	AIC	BIC	AIC	BIC	AIC	BIC
logit	148.69	159.97	147.69	158.97	156.46	167.74	-	-
probit	148.35	159.63	147.39	158.67	149.97	161.25	-	-
loglog	146.24	157.52	**145.53**	**156.81**	147.10	158.38	-	-
cloglog	-	-	-	-	-	-	-	-

Note: The AIC/BIC values, associated with the best pair of links, are highlighted in bold.

**Table A3:** Adjacent-categories mixed-link models with npo.

	logit		probit		loglog		cloglog	
	AIC	BIC	AIC	BIC	AIC	BIC	AIC	BIC
logit	148.49	159.78	147.87	159.15	146.83	158.11	149.47	160.75
probit	148.54	159.82	147.92	159.20	146.90	158.18	149.51	160.79
loglog	147.65	158.93	147.01	158.29	**145.97**	**157.25**	148.56	159.85
clog log	150.20	161.49	149.62	160.90	148.71	159.99	151.17	162.46

Note: The AIC/BIC values, associated with the best pair of links, are highlighted in bold.

**Table A4:** Continuation-ratio mixed-link models with npo.

	logit		probit		loglog		cloglog	
	AIC	BIC	AIC	BIC	AIC	BIC	AIC	BIC
logit	148.95	160.23	148.30	159.58	147.28	158.56	149.81	161.09
probit	148.55	159.84	147.91	159.19	146.88	158.16	149.41	160.70
loglog	147.61	158.89	146.96	158.24	**145.93**	**157.21**	148.47	159.75
clog log	151.95	163.23	151.30	162.58	150.27	161.55	152.81	164.09

Note: The AIC/BIC values, associated with the best pair of links, are highlighted in bold.

**Table A5:** Baseline-category mixed-link models with po.

	logit		probit		loglog		cloglog	
	AIC	BIC	AIC	BIC	AIC	BIC	AIC	BIC
logit	173.33	181.79	202.33	210.79	197.84	206.30	188.22	196.68
probit	**151.12**	**159.58**	172.76	181.22	164.81	173.27	162.85	171.31
loglog	159.04	167.50	180.71	189.17	176.63	185.09	170.09	178.55
clog log	156.83	165.29	176.00	184.46	169.15	177.61	166.43	174.89

Note: The AIC/BIC values, associated with the best pair of links, are highlighted in bold.

**Table A6:** Cumulative mixed-link models with po.

	logit		probit		loglog		cloglog	
	AIC	BIC	AIC	BIC	AIC	BIC	AIC	BIC
logit	150.23	158.70	194.90	203.36	204.72	213.18	-	-
probit	147.75	156.21	149.25	157.71	155.53	163.99	-	-
loglog	**144.29**	**152.75**	148.81	157.27	147.08	155.54	-	-
cloglog	-	-	-	-	-	-	-	-

Note: The AIC/BIC values, associated with the best pair of links, are highlighted in bold.

**Table A7:** Adjacent-categories mixed-link models with po.

	logit		probit		loglog		cloglog	
	AIC	BIC	AIC	BIC	AIC	BIC	AIC	BIC
logit	148.87	157.33	153.58	162.04	153.82	162.28	151.66	160.12
probit	146.57	155.03	148.59	157.05	148.14	156.60	148.17	156.63
loglog	**146.33**	**154.79**	149.87	158.34	149.56	158.02	148.63	157.09
clog log	148.21	156.67	150.03	158.49	149.74	158.20	149.68	158.14

Note: The AIC/BIC values, associated with the best pair of links, are highlighted in bold.

**Table A8:** Continuation-ratio mixed-link models with po.

	logit		probit		loglog		cloglog	
	AIC	BIC	AIC	BIC	AIC	BIC	AIC	BIC
logit	154.43	162.89	167.30	175.76	165.20	173.66	162.05	170.51
probit	147.39	155.85	153.58	162.04	152.13	160.59	150.99	159.45
loglog	**146.96**	**155.42**	153.40	161.86	151.96	160.43	150.83	159.29
cloglog	152.17	160.63	161.47	169.94	159.67	168.13	157.42	165.88

Note: The AIC/BIC values, associated with the best pair of links, are highlighted in bold.
